# Exploring the relationship between occupational stress, physical activity and sedentary behavior using the Job-Demand-Control Model

**DOI:** 10.3389/fpubh.2024.1392365

**Published:** 2024-10-28

**Authors:** Maëlys Clinchamps, Céline Bibily, Jean-Baptiste Bouillon-Minois, Ukadike C. Ugbolue, Marion Trousselard, Bruno Pereira, Frédéric Dutheil

**Affiliations:** ^1^CNRS, LaPSCo, Physiological and Psychosocial Stress, CHU Clermont–Ferrand, Occupational and Environmental Medicine, Université Clermont Auvergne, Clermont-Ferrand, France; ^2^Occupational and Environmental Medicine, Université Clermont Auvergne, Clermont-Ferrand, France; ^3^CNRS, LaPSCo, Physiological and Psychosocial Stress, CHU Clermont–Ferrand, Emergency Medicine, Université Clermont Auvergne, Clermont-Ferrand, France; ^4^Biomechanics Laboratory, Sport and Physical Activity Research Institute (SPARI), School of Health and Life Sciences, University of the West of Scotland, Scotland, United Kingdom; ^5^Neurophysiology of Stress, French Armed Forces Biomedical Research Institute-IRBA, Brétigny-sur-Orge, France; ^6^Clinical Research and Innovation Direction, University Hospital of Clermont Ferrand, Clermont-Ferrand, France

**Keywords:** Job-Demand-Control Model, work, occupational stress, sedentary behavior, physical activity

## Abstract

**Objectives:**

To study the relationship between the occupational stress model, specifically the Job Demand-Control Model of Karasek, physical activity level and sedentary behavior.

**Method:**

This is a cross-sectional, observational, descriptive study. A self-administered questionnaire was distributed to 100 volunteers working at Clermont Auvergne University. The questionnaire included the Karasek questionnaire and the International Physical Activity Questionnaire.

**Results:**

The results reveal that occupational characteristics play a significant role, with individuals exhibiting high job control showing reduced sitting time and increased physical activity compared to those with low job control. Job strain was associated with increased sitting time and decreased physical activity. Further analysis revealed that being in a state of job strain significantly predicted sitting for more than 7 h per day. Similarly, job strain and isostrain were explanatory factors for having a low to moderate physical activity level. Logistic regression quantified the risks, indicating that sitting for more than 7 h per day increased the risk of job strain by 4.80 times, while high physical activity levels and being male reduced the risk by 79 and 84%, respectively. Job strain also increased the risk of prolonged sitting by 5.06 times and low to moderate physical activity levels by 5.15 times. Additionally, mediation analysis revealed that a substantial portion of the association between sitting time and job strain was mediated by physical activity, and vice versa, emphasizing the interconnected nature of sedentary behavior and physical activity in influencing occupational stress.

**Conclusion:**

The study highlights the impact of sedentary behavior on occupational stress, assessed using Karasek’s Job-Demand-Control Model. Despite being less studied, sedentary behavior appears to be a relevant contributor to occupational stress. Furthermore, the results emphasize the significant role of physical activity levels, suggesting that it plays a substantial part in the relationship between sedentary behavior and occupational stress.

## 1 Introduction

Work has undergone significant changes over the past few decades, with increasing attention on employee well-being and mental health (1). One of the main shifts has been the rise of technological advancements, including digital revolution and task automation, which have boosted productivity but also contributed to a decline in manual work in favor of more sedentary jobs (2). Additionally, the integration of digital tools has transformed the nature of work, requiring employees to develop new skills and adapt to rapidly changing technologies (3). These evolutions have been modulated by social, economic, and technological factors, that have both positive and negative impacts on individuals and society as a whole (4). While physical activity is recognized for its beneficial effects on health (5), sedentary behavior has a wide range of negative impacts (6, 7). Sedentary behavior is defined as “Any waking behaviors characterized by an energy expenditure ≤1.5 METs, while in a sitting, reclining, or lying posture” (8). A sedentary lifestyle is a major contributor to preventablemortality in developed countries, with the majority of the working population spending around a third of their waking hours seated (9). Coupled with the increasing demands and pressures of the modern workplace, a sedentary lifestyle has made stress at work a common issue affecting millions globally (10). According to the WHO, stress can be defined as “a state of worry or mental tension caused by a difficult situation” while work-related stress refers to “the response people may have when presented with work demands and pressures that are not matched to their knowledge and abilities and which challenge their ability to cope.” Stress is recognized as a significant public health problem, especially when it becomes chronic and leads to adverse health outcomes (11). There are several methods for assessing stress at work, but one of the reference models is the “Job Demand-Control-Support” (JDCS) model of Karasek, that assesses psychological demand, decision latitude and social support (12–15). The combination of the two dimensions leads to the creation of four quadrants: Active (high demand, high control), where employees find their work challenging but have the resources and control to manage the demands effectively; Low-strain (low demand, high control), where jobs are less stressful, but may lack stimulation or challenge; Passive (low demand, low control), where jobs may be monotonous and less engaging, potentially leading to feelings of boredom or disinterest; and finally, High-strain (high demand, low control), where employees face high demands without sufficient control. This last quadrant reflects the work-related stress, and is also called “Job strain.” Adding low support to this quadrant leads to an “Isostrain” situation that has the highest risk of stress-related health issues, as employees face high demands, limited control, and lack social support. The impact of Job strain and Isostrain is well documented and can lead to a large number of physical and mental health problems. Stress at work is well known for its relation with mental, physical, and social well-being, including increased mortality (16–19). Simultaneous presence of prolonged sitting and stress in the workplace, generated by transitions in the world of work, has significant implications for both physical and mental well-being (20). Therefore, understanding the causes and consequences of occupational stress is crucial for developing effective interventions to improve the well-being of workers and promote a healthier and more productive workforce. One of the courses of action could lie in the relation between stress at work and the level of physical activity and sedentary behavior. Indeed, evidence exists to support the fact that physical activity, has a preventative effect on global heath and mortality (5). More specifically, practicing a physical activity would be associated with a reduction in stress levels (21, 22). The literature has also shown a negative effect of stress on the level of PA, supporting the hypothesis of a bi-directional relationship (23). Indeed, studies suggest that experiencing stress can be a significant barrier to reaching recommended physical activity levels (22). However, the relation between stress and sedentary behavior is less studied. Extended periods of sitting have a negative impact on the musculoskeletal system, which increases tension and soreness in the muscles, especially in the neck and lower back (24–26). This lack of mobility causes muscular imbalances and stiffness, which can worsen physical discomfort and have a detrimental effect on mental health and stress feeling (27, 28). However, the few existing studies on the relation between stress and sedentary behavior seem to be divergent. Some studies have shown that reducing the number of hours spent sitting each day decreases stress levels (29, 30), while other studies found no relationship between sedentary behavior and stress (31, 32). In particular, when considering Karasek model, participation in regular physical activities could help to cope with job strain (33) while physically inactivity may increase for people experiencing job strain (34). More specifically, considering the sub-dimensions of Karasek’s model, a study demonstrated that vigorous-intensity physical activity and sedentary behavior were negatively associated with demand and support (35). Although physical activity appears to have a protective effect against stress, the relationship between sedentary behavior and stress, especially when assessed using the JDCS model, remains unclear and requires further research to fully understand the mechanisms between these factors. Moreover, each profession has its own specific characteristics in terms of physical activity and sedentary lifestyles, which means that risk prevention policies need to be adapted accordingly. University staff encompass a wide range of occupations, from highly sedentary to highly active roles, allowing for the study of a more representative sample of profiles. Additionally, research suggests that teachers who engage in regular physical activity are more likely to promote physical activity among their students, thereby creating a culture of health within educational settings (36). Therefore, the aim of this article is to provide a comprehensive overview of the relationship between work-related stress as evaluated using the JDCS model, physical activity, and sedentary behavior.

## 2 Method

### 2.1 Study design

We conducted a cross-sectional, observational, descriptive study. We used data from a self-administered questionnaire collected in a 2017 survey conducted by our Occupational Medicine department, which was offered to workers at the Université Clermont Auvergne in Occupational health consultations. There were no inclusion or exclusion criteria in this study. All data were anonymous. This study was approved by the ethics committee South-East I (Comité de Protection des Personnes Sud-Est VI, Clermont-Ferrand, France; No. 2015/CE 70).

### 2.2 Participants

The participants were university staff from the administrative, technical and teaching fields.

### 2.3 Outcomes

The self-administered questionnaire comprised two validated questionnaires and sociodemographic variables:

The Job Demand Control-Support model (JDCS) of Karasek assesses the psychological demands of the job, the level of decision-making authority granted to workers, and the social support they receive (13). We used the validated French version of the questionnaire, which demonstrated satisfactory internal consistency with Cronbach’s alpha coefficients exceeding the acceptable threshold of 0.70 for its scales measuring decision latitude and psychological demand (14). The JDCS comprises 26 questions, with nine questions related to job demand, nine related to job control, and eight related to social support. The questions are rated on a 4-point Likert scale, ranging from 1 (“strongly disagree”) to 4 (“strongly agree”). A worker with a low latitude score (below 71) and a high demand score (above 20) is considered to experiencing job strain or tension at work. Among those workers with job strain, a low support score (below 24) represents an additional psychosocial risk factor that characterizes “isostrain” (14).

The International Physical Activity Questionnaire (IPAQ) (37) assesses the overall physical activity and the level of sedentary lifestyle during the previous 7 days. We used the French version of the questionnaire which presents a majority of Spearman correlation coefficients around 0.8, indicating very good internal consistency (37). The 27 items evaluate the practice of intense or moderate activities, walking, as well as time spent sitting (sedentary), whether during leisure, work, or transport. Data collected with IPAQ can be reported as a continuous measure (metabolic equivalent task (MET)-minutes per week) for physical activity, and time spent sitting expressed in number of minutes per day on weekdays for sedentary behavior. For qualitative analysis, sedentary behavior was categorized into two levels based on the median value as recommended in the IPAQ Guidelines for Data Processing and Analysis (≤7 h/d vs. >7 h/d) and physical activity was classified as low/moderate vs. high.

We also collected sociodemographic data, including age, gender, marital status, parenthood and occupation. Age was a continuous variable, dichotomized at the mean value of 42 years. Gender was a categorical variable with categories: male and female.

### 2.4 Statistics

Categorical data are presented as numbers (*n*) and percentages (%), while quantitative data are expressed as mean ± standard deviation (SD).

The sedentary behavior, physical activity and JDCS model variables were first treated as continuous and then categorized into binomial variables as follows: sitting time: ≤7 h/day / >7 h/day; (physical activity: “low/moderate” vs. “high”; JDCS model: “job strain” vs. “no job strain”).

Spearman correlation coefficient were estimated to examine the relationship between the aforementioned variables treated as continuous. Chi-square tests were used to compare categorical variables between groups. Non-parametric statistical tests were used to compare quantitative variables between groups: Mann–Whitney test was used to compare two groups. When appropriate (omnibus *p*-value less than 0.05), Dunn’s test was used to perform multiple pairwise comparisons following the Kruskal Wallis test. The results were expressed as effect-sizes with *r* 95% Confidence Intervals (CIs), and interpreted according to Cohen’s rule of thumb: ignored when <0.20, Small <0.50, Moderate <0.80 and Large>0.80.

We then performed multivariate logistic regression analyses to assess the relationship between work-related stress as measured using the JDCS model, physical activity, and sedentary behavior. The results were reported as relative risks (RR) with 95% CIs.

Finally, two mediation analyses were conducted to assess: (1) the contribution of physical activity level to the relationship between sitting time and job strain; and (2) the contribution of sitting time to the relationship between physical activity and job strain. A mediation proportion was estimated, indicating the extent to which the relationship with the independent variable could be explained by the indirect effect of a mediator (1. Physical activity; 2. Sitting time), and how changes in the mediator then affect the outcome. Results were summarized using a graph displaying the mediation proportion and significance of the mediation analysis associations. Coefficients a, b and c are coefficient regressions and standard errors (in parentheses). The c coefficient represents the direct effect. The a and b coefficients represent the indirect effects. Statistical analysis was performed using Stata software, version 15.0 (StataCorp, College Station, TX, USA). Statistical significance was set at *p* < 0.05.

## 3 Results

**Figure 1:**
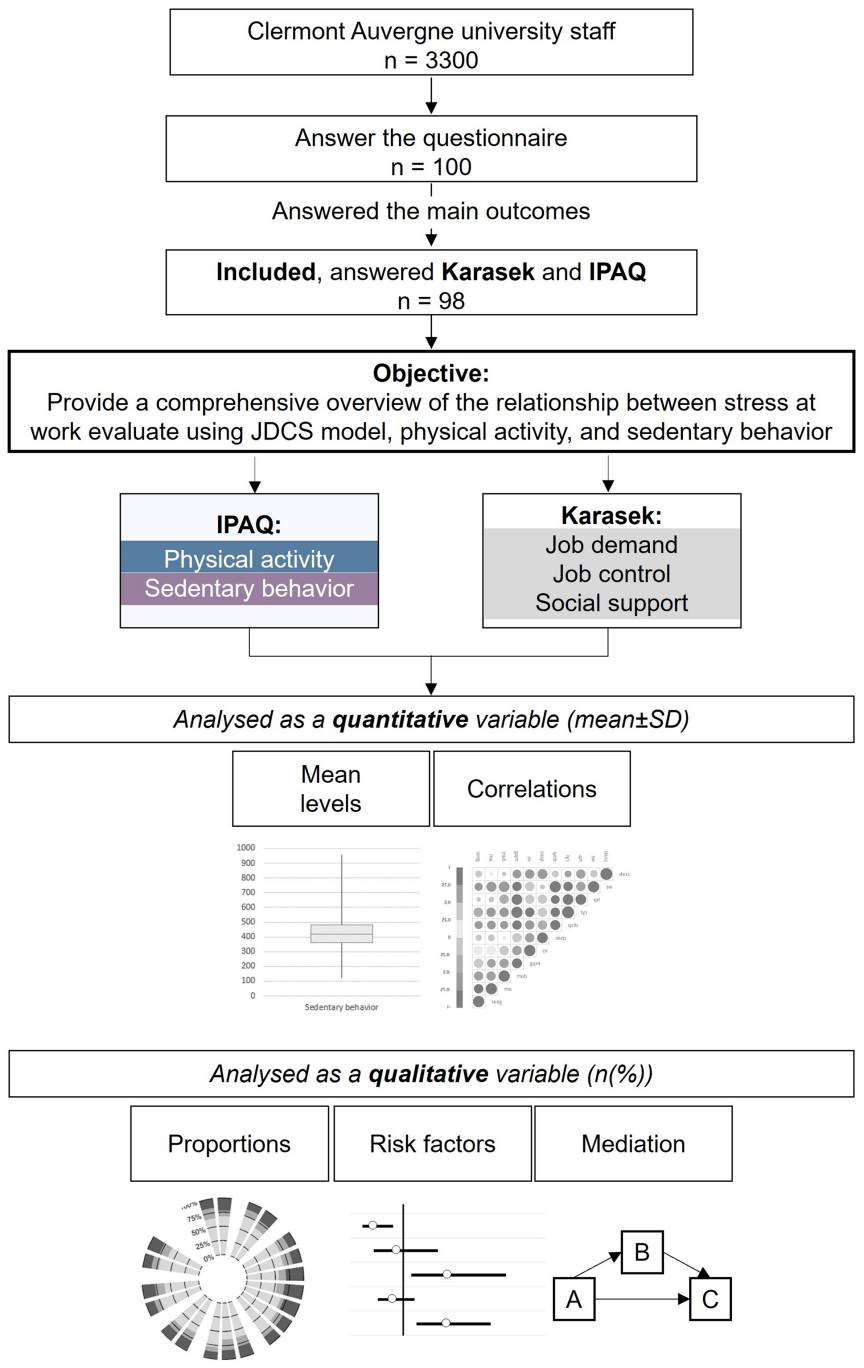
Flow chart.

**Figure 2:**
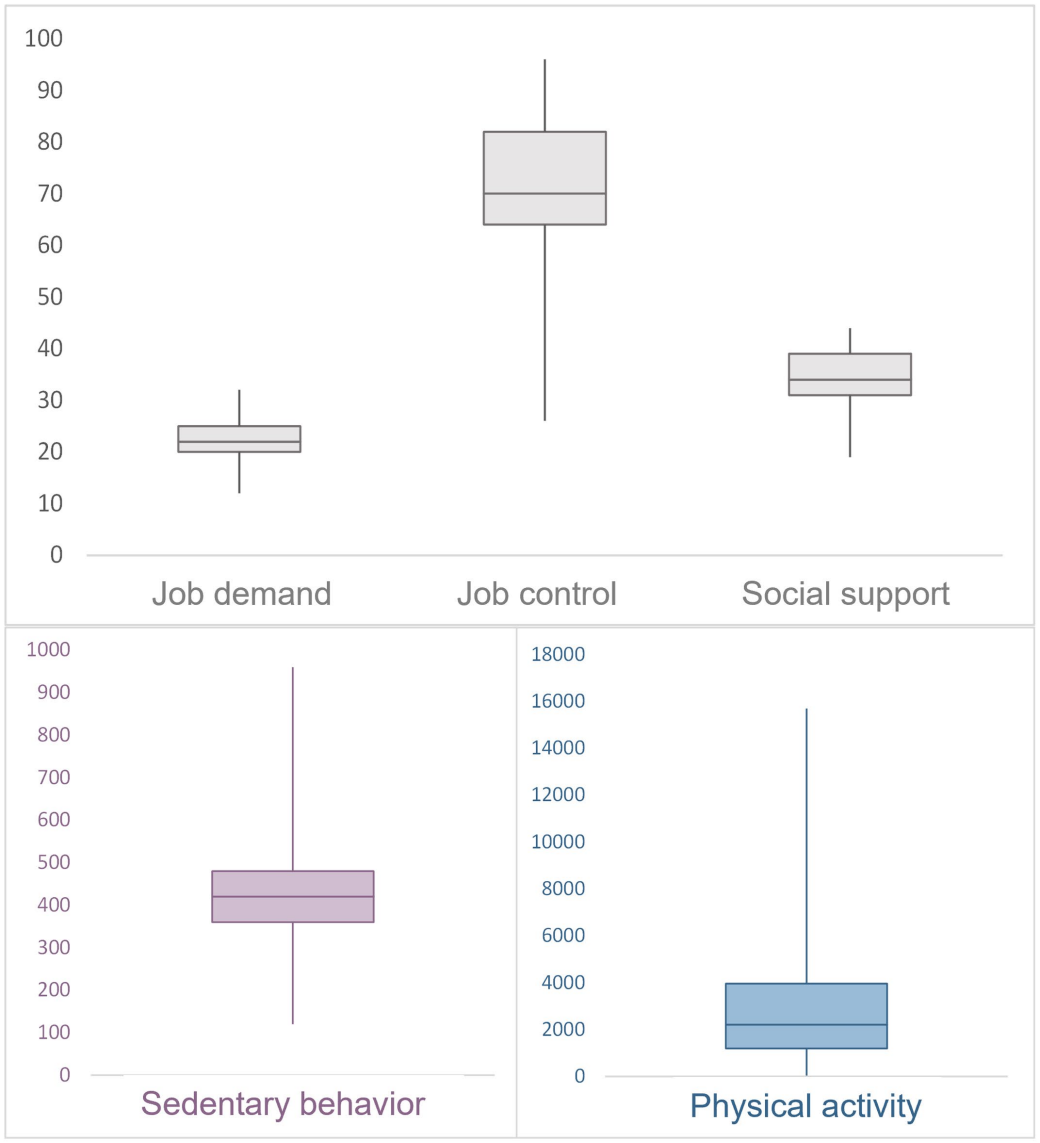
Box plot—Mean score of JDCS model sub-dimension, physical activity and sedentary behavior. Job demand, job control and social support are expressed on a scale ranging from 0 to 100. Physical activity is expressed in MET-minutes per week and sedentary behavior in number of minutes sitting per day on weekdays.

A total of 100 workers at Clermont Auvergne University responded the questionnaire, and 98 participants were included in the statistical analysis (Figure 1). The mean age of the participants was 41.3 ± 10.5 and 54.1% were women. The majority were in a relationship (67.7%) and had at least one child (60.2%). Almost half were administrative staff (46.5%) and a quarter were teachers. The mean score for job demand, job control and social support were: 22.5 ± 4.0, 71.4 ± 13.7 and 34.3 ± 5.7, respectively. The average sitting time per day during daywork was 428.9 ± 171.1 min and the average physical activity was 3480.4 ± 3559.9 MET-min per week (Figure 2).

### 3.1 Descriptive analysis—sedentary behavior and physical activity as quantitative variables

#### 3.1.1 Sociodemographic variables

Age and gender did not influence time spent sitting per day during daywork or the weekly physical activity level.

#### 3.1.2 Occupational characteristics

**Figure 3:**
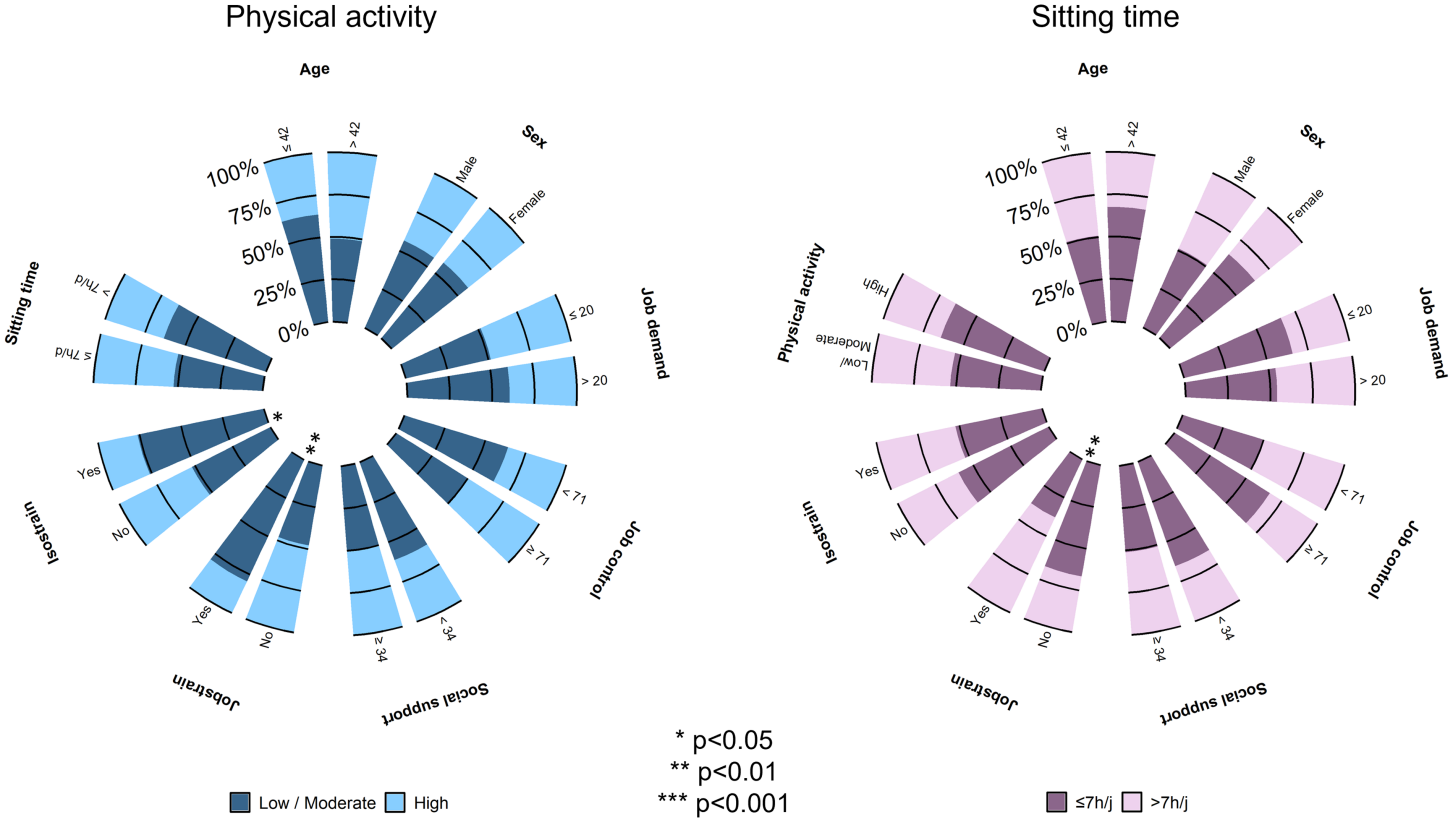
Polar plot—Prevalence of physical activity and sitting time depending on sociodemographic and job-demand-control-support model of Karasek (groups were compared using Chi-square; see Supplementary Table 1).

Sitting time and physical activity were significantly correlated (*p* < 0.001), and both were correlated to job strain (*p* = 0.002 and *p* = 0.009, respectively). However, only physical activity was correlated with job control (*p* = 0.034) (Figure 3).

Workers with high job control spent less time sitting than those with low control (392.5 ± 175.0 vs. 463.8 ± 161.4 min/day, *p* = 0.039, ES = 0.43, 95%CI 0.02 to 0.82) and tend to spend more time doing physical activity (3956.6 ± 3788.5 vs. 3023.2 ± 3299.2 MET-min/week, *p* = 0.027, ES = -0.26, −0.66 to 0.14). Similarly, those experiencing job strain spent more time sitting than those without job strain (505.9 ± 110.5 vs. 396.5 ± 167.9 min/day, *p* = 0.002, ES = -0.66, −1.11 to-0.22) and tended to spend less time doing physical activity (2561.9 ± 3210.3 vs. 3866.4 ± 3650.0 MET-min/week, *p* = 0.002, ES = −0.37, −0.09 to 0.81). Job demand, social support and isostrain did not influence time spent sitting or physical activity.

#### 3.1.3 Physical activity

Physical activity did not influence time spent sitting, but sitting time higher than 7 h per day was the only variable significantly reducing physical activity (2631.1 ± 451.8 vs. 4091.2 ± 3881.4 MET-min/week, *p* < 0.001, ES = 0.42, 0.01 to 0.82) (Table 1).

**Table 1:** Descriptive table of characteristics for quantitative variables.

*n*		Sedentary behavior on daywork Quantitative variable (min sitting/day)			Physical activity Quantitative variable (MET-min/week)		
		Mean ± SD	*p*-value	Effect size	Mean ± SD	*p*-value	Effect size
Sociodemographic							
Age							
≤42	55	446.2 ± 170.4	0.21	0.23	3,212 ± 3,678	0.21	−0.17
>42	43	406.7 ± 171.5		[−0.17; 0.63]	3,623 ± 3,414		[−0.57; 0.23]
Sex							
Male	45	456 ± 197.1	0.16	−0.29	3,283 ± 3,453	0.77	0.10
Female	53	405.8 ± 143.5		[−0.69; 0.10]	3,647 ± 3,672		[−0.30; 0.50]
Occupational characteristics							
Job demand							
≤20	35	414.9 ± 172.4	0.31	−0.13	3,754 ± 3,867	0.31	0.12
>20	63	436.7 ± 171.3		[−0.54; 0.29]	3,328 ± 3,399		[−0.29; 0.53]
Job control							
<71	50	463.8 ± 161.4	0.039	0.43	3,023 ± 3,299	0.027	−0.26
≥71	48	392.5 ± 175.0		[0.02; 0.82]	3,956 ± 3,788		[−0.66; 0.14]
Social support							
<34	49	405.9 ± 153.6	0.15	−0.27	3,412 ± 3,602	0.15	−0.04
≥34	49	451.8 ± 185.8		[−0.67; 0.13]	3,548 ± 3,553		[−0.43; 0.36]
Job strain							
No	69	396.5 ± 167.9	0.002	−0.66	3,866 ± 3,650	0.002	0.37
Yes	29	505.9 ± 155.8		[−1.11; −0.22]	2,561 ± 3,210		[−0.07; 0.81]
Isostrain							
No	77	421.6 ± 184.2	0.26	−0.20	3,663 ± 3,593	0.26	0.24
Yes	21	455.7 ± 110.5		[−0.68; 0.28]	2,807 ± 3,432		[−0.24; 0.72]
Lifestyle							
Physical activity							
Low/Moderate	56	454.8 ± 182.8	0.075	0.36			
High	42	394.3 ± 149.4		[−0.05; 0.76]			
Sitting time							
≤7 h/d	57				4,091 ± 3,881	<0.001	0.42
>7 h/d	41				2,631 ± 451		[0.01; 0.82]

ES are considered: Ignored when <0.20, Small <0.50, Moderate <0.80 and Large>0.80 for Cohen’s d.

### 3.2 Descriptive analysis—sedentary behavior and physical activity as qualitative variables

**Figure 4:**
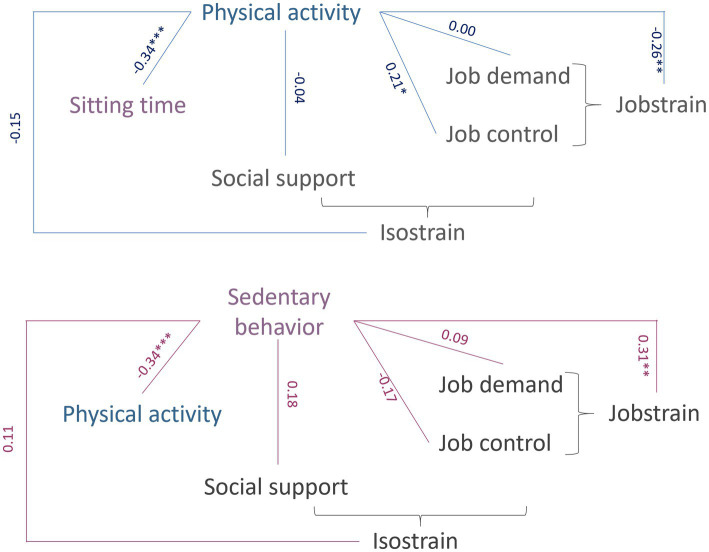
Spearman correlation analysis between physical activity, sedentary behavior and job-demand-control-support model of Karasek.

When sedentary behavior was considered as a categorial variable, being in job strain was the only significant factor associated with sitting for more than 7 h per day (*p* = 0.008, ES = 0.27). Regarding physical activity as a categorial variable, being in job strain or isostrain was an explanatory factor for having a low to moderate physical activity level (*p* = 0.004, ES=NS et *p* = 0.047, ES=NS, respectively) (Figure 4).

### 3.3 Risk quantification—multivariate analysis

Sitting for more than 7 h per day was the only factor that increased the risk of job strain, multiplying the risk by 4.80 (95% CI 1.63 to 14.1, *p* = 0.004). In contrast, a high level of physical activity reduced the risk of job strain by 79% (RR = 0.21, 95% CI 0.07 to 0.65, *p* = 0.007) and being a male reduced the risk by 84% (RR = 0.16, 95% CI 0.05 to 0.49, *p* = 0.001). Age did not significantly influence job strain.

Being male reduced the risk of isostrain by 82% (RR = 0.18, 95% CI 0.05 to 0.61, *p* = 0.006) and having a high level of physical activity tended to reduce the risk of isostrain by 67% (RR = 0.33, 95% CI 0.10 to 1.05, *p* = 0.060). Neither age nor sitting for more than 7 h per day significantly influenced isostrain.

Job strain multiplied the risk of sitting for more than 7 h per day by 5.06 (95% CI 1.71 to 15.1, *p* = 0.003), and being a male increased the risk by 2.87 (95% CI 1.06 to 7.3, *p* = 0.032). Age, isostrain, and physical activity did not significantly influence sitting time per day.

**Figure 5:**
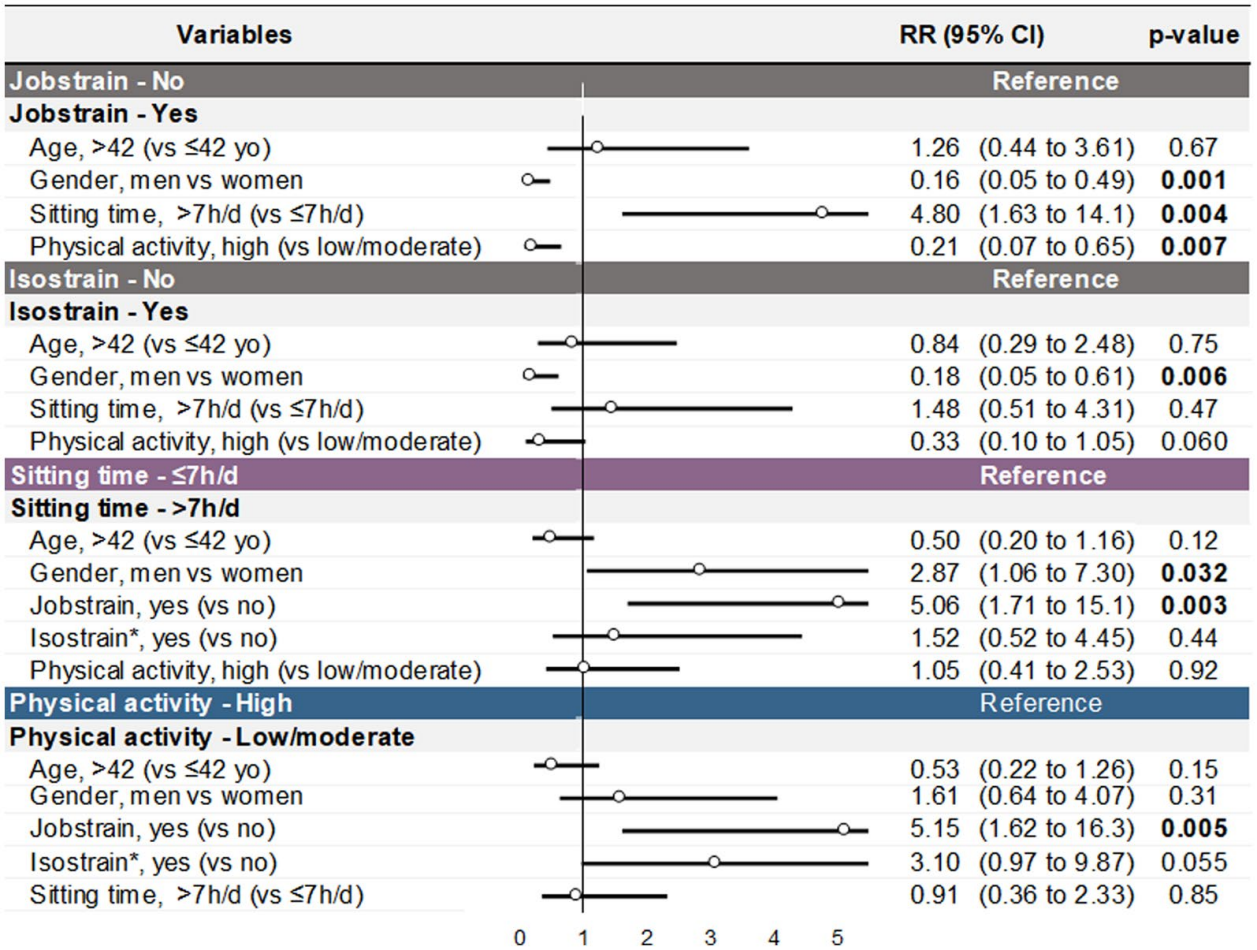
Quantification of the risk between jobstrain, sitting time ≥ 7 h/day and low/moderate physical activity level controlled on age and gender. *Multivariate analysis were computed with jobstrain. Iso and jobstrain was assessed in separate modele because of colinearity. Coefficient were similars in other variables using isostrain except for sex in sitting time (see Supplementary Figure 1).

Finally, job strain increased the risk of having a low or moderate level of physical activity by 5.15 (95% CI 1.62 to 16.3, *p* = 0.005), and isotrain tended to increase the risk by 3.10 (95% CI 0.97 to 9.87, *p* = 0.055). Age, gender, and sitting time did not significantly influence physical activity (Figure 5).

### 3.4 Mediation analysis

**Figure 6:**
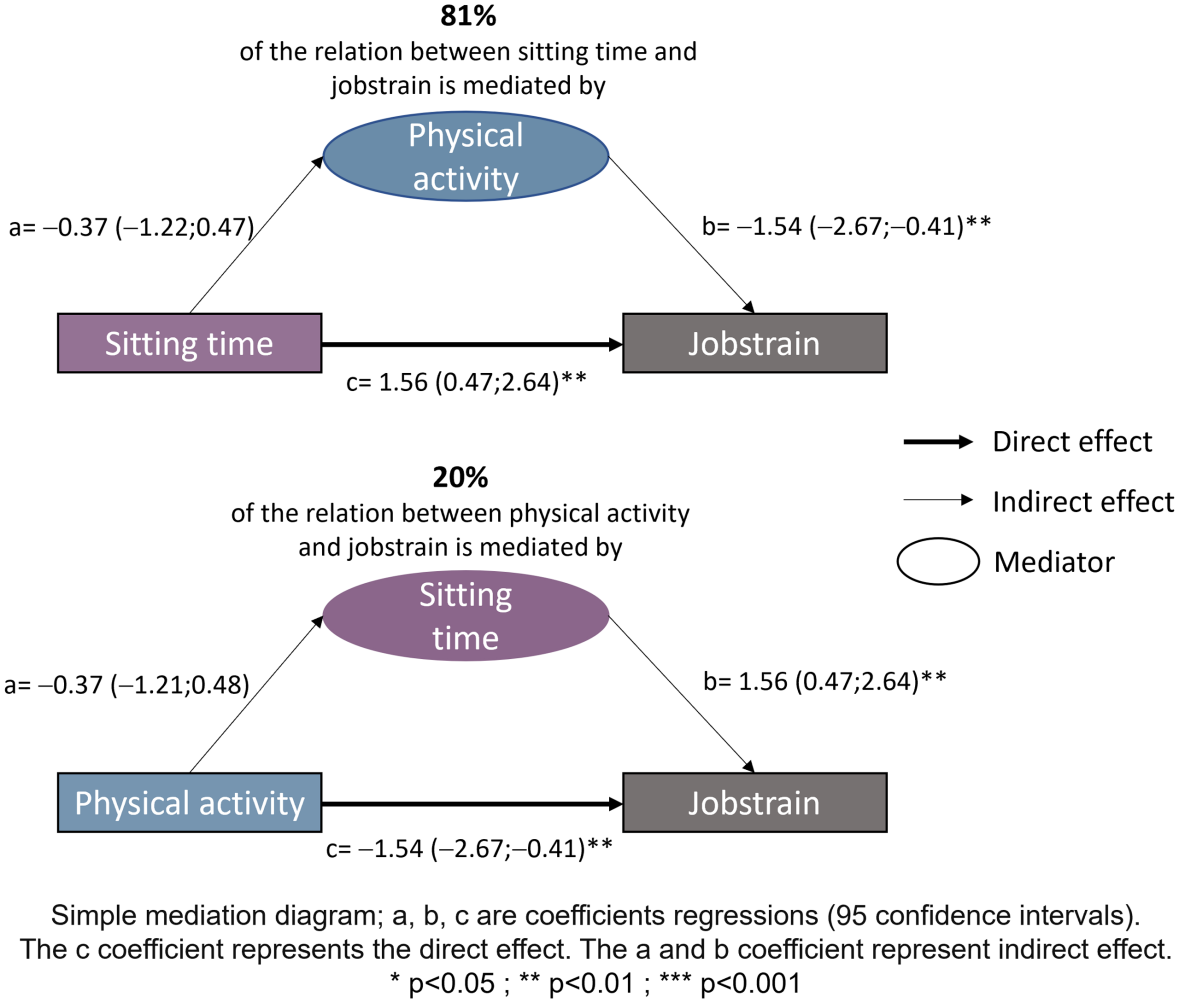
Simple mediation analysis. Simple mediation diagram, a, b, c are coefficients regressions (95 confidence intervals). The c coefficient represents the direct effect. The a and b coefficient represent indirect effect. *p < 0.05; **p < 0.01; ***p < 0.001.

Mediation analysis evaluated two pathways: (1) whether sitting time mediated the effect of physical activity on job strain; and (2) whether physical activity level mediated the effect of sedentary behavior on job strain. The analyses showed that 81% of the association between sitting time per day and job strain was mediated by physical activity level, while 20% of the association between physical activity level per week and job strain was mediated by sitting time. All relationships explored by mediation analyses are presented in Figure 6.

## 4 Discussion

The main findings were that, although less studied, sedentary behavior has a significant influence on occupational stress, as assessed using Job-Demand-Control Model of Karasek. Nevertheless, physical activity level seems to be the factor with the most weight, considering its mediation effects.

### 4.1 Occupational characteristics—sub-dimensions of Karasek’s model

Regarding our results and considering the sub-dimensions of Karasek’s model, we showed that high job control contributed to reducing sitting time and increasing physical activity. Several studies support this association. Jobs with limited decision latitude may involve tasks that require continuous sitting or minimal movement, contributing to a sedentary work environment. Conversely, occupations with greater job control may offer employees the opportunity to vary their tasks and incorporate movement, reducing sedentary time (34, 38). Thus, greater control and higher levels of decision latitude at work were protective factors against physical inactivity among both men and women (39) and were associated with higher leisure time physical activity (38, 40, 41). On the other hand, we found no influence of job demand or social support on time spent sitting and physical activity. However, some studies have shown that vigorous-intensity physical activity and sedentary behavior were negatively associated with demand and support (35). A large longitudinal study showed that social support at work was a protective factor against physical inactivity among women (39). It has been suggested that the impact of high job demands on physical activity may depend on the level of job control available to individuals (42). A study found that workers with sufficient job control to accomplish the required tasks may not experience a negative impact on their physical activity despite having a highly demanding job (42). Employees with a higher degree of autonomy in their work might benefit from greater flexibility to arrange their work hours, facilitating their participation in physical activity (34). These results suggest that the level of job control could mitigate the adverse effects of elevated job demands on physical activity. Studies on the relationship between the Job Demand-Control-Support model and objectively measured physical activity and sedentary time have yielded data showing that a reduction in sedentary time and an increase in physical activity may be beneficial in managing high-demand and low-support work environments (35).

### 4.2 Occupational characteristics—job strain

As found in the literature, we showed a strong bidirectional relationship between job strain and physical activity. The association between job strain and physical activity levels presents a complex relationship. Job strain not only affects physical activity, but the levels of physical activity can also influence how individuals perceive and manage job strain (43). Several studies have reported a negative impact of high-strain jobs on the overall physical activity level (44). For example, a study on female and male public sector employees found that high job strain was associated with lower leisure-time physical activity (2.6 to 5.2 MET-hours/week less), even after adjusting for various factors (45). A field study using a consumer fitness tracker found that high-demand work environments (high job demands and low job control) were associated with less leisure time physical activity (46). The likelihood of physical inactivity or engaging in physical activities for less than the recommended duration was found to be higher in individuals experiencing job strain (39). Furthermore, a meta-analysis involving as many as 170,000 men and women revealed that individuals employed in high-strain jobs had a 26% increased likelihood of being physically inactive during leisure time, in contrast to their counterparts in low-strain jobs (marked by high control and low demands) (34). On the other hand, few studies have shown a weak (47) or even non-existent association (48). We also showed a bidirectional relationship between job strain and sedentary behavior. Nonetheless, this relationship is less studied in the literature, as sedentary behavior is often confused with physical inactivity. Existing studied seems to confirm this relationship. Indeed, a cross-sectional study of 6,995 white-collar workers found that job strain was associated with sedentary behavior. Sedentary behavior was elevated in women with the highest quartile of psychological demands (49). Job strain may contribute to sedentary behavior, as individuals may cope with stress by engaging in passive activities such as watching TV or spending more time sitting (34). These findings collectively suggest that job strain may contribute to an increase in sedentary behavior among workers.

### 4.3 Sociodemographic and other variables

In our study on active adults, we found no relationship between age and sex with physical activity level and sitting time but the literature shows strong relationships between them. Many studies highlight gender differences in physical activity levels. Men are found to be more physically active than women, particularly in terms of engaging in vigorous activities. For instance, a study involving 116,982 adults highlighted the differences in physical activity behavior between men and women (50). The activity levels of women are comparatively lower than those of men, as evidenced by the global averages indicating that 31.7% of women are inactive, in contrast to 23.4% of men (51). This disparity is observed across various age groups and contexts. Indeed, a study found that, from a young age, both in children and adolescents, boys showed a higher prevalence of meeting physical activity guidelines compared to girls, with a gender difference of over 8% in favor of boys (52). Furthermore, studies consistently show a decline in physical activity levels with age. As individuals progress through the different stages of life, physical exercise levels can exhibit considerable variability, with a general decline observed from childhood to older adulthood (53). Older adults tend to engage in less moderate to vigorous physical activity compared to younger individuals and few older adults meet minimum guidelines for physical activity (54, 55) Sedentary behavior patterns may also differ by sex. Research findings on whether women are more sedentary than men may depend on the specific context of the studied population. Women may be more likely to have sedentary jobs, such as office-based work, which could contribute to higher overall sedentary time during working hours (56). Moreover, sedentary behavior tends to increase with age. Older adults often spend more time sitting or engaging in low-energy activities. Research has shown that sedentary behavior is highly prevalent in older adults and is associated with poor physical function, which can lead to accelerated skeletal muscle aging (57). Additionally, a study on the transition from working life to retirement found that retirement was associated with increased sedentary time (58).

### 4.4 Limitations

Several limitations were noted in this study. First, this study was mono-centric, on a limited number of subjects, which may not be representative of the population as a whole. Therefore, results from our study may not be generalizable to other population or contexts. Furthermore, the cross-sectional design posed challenges in establishing causal relation (59). Our study exclusively uses self-reported data, allowing each participant to respond anonymously without any means of verification. Nevertheless, the use of anonymity might have served as a deterrent against potential misreporting. Furthermore, we used the International Physical Activity Questionnaire (IPAQ) to assess physical activity level and sedentary behavior. Although the small number of respondents to the IPAQ may be a limitation, there is no minimum number of subjects required to administer the IPAQ. A recent meta-analysis on the validity on questionnaires assessing physical activity found that more than half of the studies included less than 100 participants (60). Although the IPAQ is widely used and can provide measurements of time spent in specific sedentary behaviors, it is subject to recall bias, usually resulting in an overestimation of physical activity and an underestimation of sedentary behaviors (61, 62). Furthermore, it showed moderate validity against objective measures such as accelerometers (63). However, accelerometers are also subjected to bias (61). On the other hand, our study highlighted the complex interconnection between sedentary lifestyle, physical activity and work stress. Unlike physical activity, there are no clear recommendations on the amount of time spent sitting or on what constitutes a risk. The WHO only recommends limiting the amount of time spent being sedentary, without specifying limits (64). Although the data suggest a dose–response effect between sitting time and health outcomes, there is insufficient evidence to make a time-based recommendation, particularly given the considerable variation in how sedentary behavior is assessed and the likelihood that sedentary time thresholds may vary across physical activity levels or population subgroups (65). In the same area, our research team previously proposed the use of the term sedentariness to define sedentary lifestyle. Indeed, sedentary behavior describes actions taken at a particular moment without offering any long-term details. Moreover, identifying sedentariness as a way of life is a necessary first step before making recommendations (66).

Overall, the results presented in this article suggest that reducing sedentary behavior, promoting physical activity among workers, and integrating holistic approaches to manage stress at work are effective ways to improve overall health outcomes and well-being. Further research and attention to these factors can contribute to the development of targeted strategies aimed at improving overall employee well-being and productivity in the workplace. Lastly, there is also a need for multicomponent evaluations of stress and sedentary behavior. Conveniently, the use of smart technology is now able to measure biomarkers of stress such as heart rate variability and accelerometry (67–69). It would be of particular interest to better understand the complex interplay between all types of stress at work [mental (70), physical (71), sleep deprivation (72), insufficient food intake (73), psychosocial context (74), characteristics of sedentary behavior (75), and biological responses (76–78)].

## 5 Conclusion

In conclusion, the study highlights the impact of sedentary behavior on occupational stress in university staff members, as assessed through the Job-Demand-Control Model of Karasek. Despite being less explored in comparison to other factors, the findings underscore the significance of recognizing sedentary behavior as a relevant contributor to occupational stress. Furthermore, the results emphasize the main role of physical activity levels, suggesting that it holds substantial weight in mediating the relationship between sedentary behavior and occupational stress. This insight could provide valuable implications for workplace interventions and policies, encouraging a comprehensive approach that addresses both sedentary behavior and physical activity to mitigate occupational stress effectively. Further research on others population could contribute to the better understanding relationship between stress at work, physical activity, and sedentary behavior.

## Data Availability

The raw data supporting the conclusions of this article will be made available by the authors, without undue reservation.
