# Supplementary material for: Exploring the relationship between occupational stress, physical activity and sedentary behavior using the Job-Demand-Control Model
Source: Front Public Health. 2024 Oct 28;12:1392365. doi: 10.3389/fpubh.2024.1392365 (PMC11551119; doi:10.3389/fpubh.2024.1392365)

Supplementary Material

**Supplementary Table 1.** Descriptive table of characteristics for qualitative variables

*ES are considered: Ignored when <0.20, Small <0.50, Moderate <0.80 and Large>0.80 for Cohen’s d.* * NS : non-significant

|  | **Sedentary behavior on daywork** | | | |  | **Physical activity** | | | |
| --- | --- | --- | --- | --- | --- | --- | --- | --- | --- |
|  | **Qualitative variable** | | | |  | **Qualitative variable** | | | |
|  | **≤7h/j** | **>7h/j** |  |  |  | **Low / Moderate** | **High** |  |  |
|  | **n**  **(%)** | | **P**  **-value** | **Effect**  **size** |  | **n**  **(%)** | | **p-value** | **Effect size** |
| **Sociodemographic** | |  |  |  |  |  |  |  |  |
| **Age** |  |  |  |  |  |  |  |  |  |
| ≤ 42 | 28 (50.9) | 27 (49.1) | 0.10 | NS |  | 35 (63.6) | 20 (36.4) | 0.14 | NS |
| > 42 | 29 (67.4) | 14 (33.6) |  |  |  | 21 (48.8) | 22 (51.2) |  |  |
| **Sex** |  |  |  |  |  |  |  |  |  |
| Male | 23 (51.1) | 22 (48.9) | 0.19 | NS |  | 30 (56.6) | 23 (43.4) | 0.91 | NS |
| Female | 34 (64.2) | 19 (35.8) |  |  |  | 26 (57.8) | 19 (42.2) |  |  |
|  |  |  |  |  |  |  |  |  |  |
| **Occupational characteristics** | |  |  |  |  |  |  |  |  |
| **Job demand** |  |  |  |  |  |  |  |  |  |
| ≤ 20 | 23 (65.7) | 12 (34.3) | 0.26 | NS |  | 18 (51.4) | 17 (48.6) | 0.39 | NS |
| > 20 | 34 (54.0) | 29 (46.0) |  |  |  | 38 (60.3) | 25 (39.7) |  |  |
| **Job control** |  |  |  |  |  |  |  |  |  |
| < 71 | 25 (50) | 25 (50) | 0.095 | NS |  | 32 (64.0) | 18 (36.0) | 0.16 | NS |
| ≥71 | 32 (66.7) | 16 (33.3) |  |  |  | 24 (50.0) | 24 (50.0) |  |  |
| **Social support** |  |  |  |  |  |  |  |  |  |
| < 34 | 32 (65.3) | 17 (34.7) | 0.15 | NS |  | 30 (61.2) | 19 (38.8) | 0.41 | NS |
| ≥ 34 | 25 (51.0) | 24 (49.0) |  |  |  | 26 (53.1) | 23 (46.9) |  |  |
| **Jobstrain** |  |  |  |  |  |  |  |  |  |
| No | 46 (66.7) | 23 (33.3) | **0.008** | 0.27 |  | 33 (47.8) | 36 (52.2) | **0.004** | NS |
| Yes | 11 (37.9) | 18 (62.1) |  | [0.08; 0.45] |  | 23 (79.3) | 6 (20.7) |  |  |
| **Isostrain** |  |  |  |  |  |  |  |  |  |
| No | 46 (59.7) | 31 (40.3) | 0.55 | NS |  | 40 (52.0) | 37 (48.0) | **0.047** | NS |
| Yes | 11 (52.4) | 10 (47.6) |  |  |  | 16 (76.2) | 5 (23.8) |  |  |
|  |  |  |  |  |  |  |  |  |  |
| **Lifestyle** |  |  |  |  |  |  |  |  |  |
| **Physical activity** |  |  |  |  |  |  |  |  |  |
| Low / Moderate | 30 (53.6) | 26 (46.4) | 0.29 | NS |  |  |  |  |  |
| High | 27 (64.3) | 15 (35.7) |  |  |  |  |  |  |  |
| **Sitting time** |  |  |  |  |  |  |  |  |  |
| ≤7h/d |  |  |  |  |  | 30 (52.6) | 27 (47.4) | 0.29 | NS |
| >7h/d |  |  |  |  |  | 26 (63.4) | 15 (36.6) |  |  |
|  |  |  |  |  |  |  |  |  |  |

**Supplementary Figure 1.** Quantification of the risk between isostrain, sitting time ≥7h/day and low/moderate physical activity level controlled on age and gender.

**Multivariate analysis was computed with isostrain. Iso and jobstrain was assessed in separate model because of collinearity. Coefficient were similar in other variables using jobstrain except for sex in sitting time*


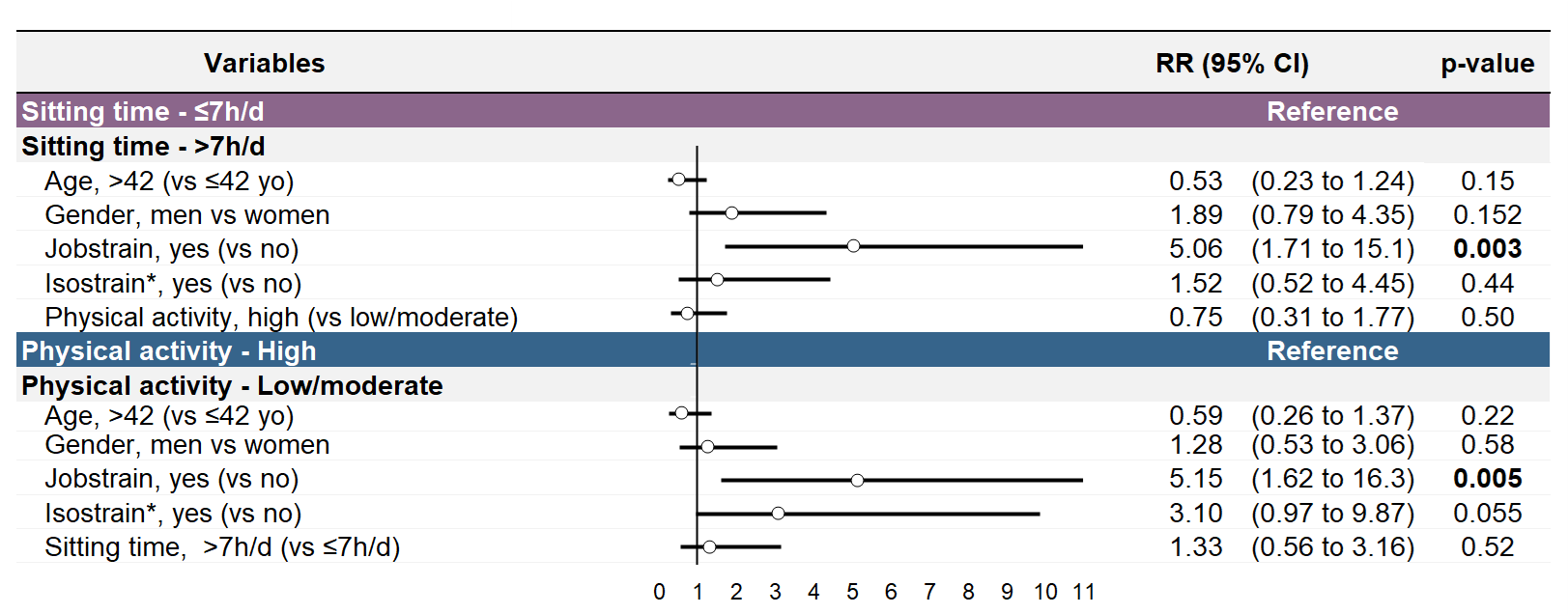

Supplement: Supplementary file 1 [file Data_Sheet_1.docx]
